# Whole-Genome Sequencing-Based Characterization of *Clostridioides difficile* Infection Cases at the University Hospital Centre Zagreb

**DOI:** 10.3390/microorganisms12122434

**Published:** 2024-11-27

**Authors:** Marko Siroglavic, Paul G. Higgins, Lucija Kanizaj, Ivana Ferencak, Dragan Juric, Goran Augustin, Ana Budimir

**Affiliations:** 1 Department of Clinical Microbiology, Infection Prevention and Control, University Hospital Centre Zagreb, Kispaticeva st. 12, 10000 Zagreb, Croatia; msirogla@kbc-zagreb.hr (M.S.); lucija.kanizaj@kbc-zagreb.hr (L.K.); ana.budimir@kbc-zagreb.hr (A.B.); 2 Institute for Medical Microbiology, Immunology and Hygiene, University of Cologne, Goldenfelsstraße 19-21, 50935 Cologne, Germany; paul.higgins@uni-koeln.de; 3 German Centre for Infection Research (DZIF), Partner Site Bonn-Cologne, 50935 Cologne, Germany; 4 Department of Microbiology, Croatian Institute of Public Health, Rockefeller st. 7, 10000 Zagreb, Croatia; ivana.ferencak@hzjz.hr (I.F.); dragan.juric@hzjz.hr (D.J.); 5 Department of Surgery, University Hospital Centre Zagreb, Kispaticeva st. 12, 10000 Zagreb, Croatia; 6 Department of Microbiology and Parasitology, School of Medicine, University of Zagreb, 10000 Zagreb, Croatia

**Keywords:** intra-hospital transmission, *Clostridioides difficile*, whole-genome sequencing

## Abstract

We investigated the intra-hospital distribution of *C. difficile* strains by whole-genome sequencing (WGS) of isolates collected in 2022 at the University Hospital Centre (UHC) Zagreb. In total, 103 patients with first-episode CDI in 2022 at UHC Zagreb were included, based on the screening stool antigen test for GDH (RidaQuick CD GDH; R-Biopharm AG, Germany), confirmed by Eazyplex *C. difficile* assays (Eazyplex CD assay; AmplexDiagnostics GmbH, Germany) specific for A, B, and binary toxins. Demographic and clinical data were retrospectively analyzed from electronic medical records. All samples were subjected to WGS analysis. Genetic clusters were formed from isolates with no more than six allelic differences according to core genome MLST. We identified six clusters containing 2–59 isolates with 15 singletons and 30 instances of possible intra-hospital transmission, mostly in the COVID-19 ward. WGS analysis proved useful in identifying clusters of isolates connecting various patient wards with possible transmission routes in the hospital setting. It could be used to support local and national surveillance of CDI infections and their transmission pathways.

## 1. Introduction

*Clostridioides difficile* (formerly known as *Clostridium difficile*) is acknowledged as a major cause of healthcare-associated infections (HAIs) and contributes to significant rates of morbidity and mortality [1,2]. *C. difficile* infection (CDI) has become an increasing concern globally due to its high recurrence and substantial economic burden [3,4,5,6]. Although *C. difficile* is mostly a nosocomial infection, more reports of CDI are being recognized as community-associated, with half of the cases remaining undiagnosed [7,8].

Virulence factors responsible for many CDI characteristics are enterotoxin (TcdA) and cytotoxin (TcdB). Some strains produce *C. difficile* transferase (CDT)—a binary toxin believed to be responsible for epidemic outbreaks of severe disease [9,10].

The ability of *C. difficile* to form spores contributes to its resistance to common disinfectants used in healthcare settings, as well as alcoholic hand rubs. This resistance facilitates these bacteria’s horizontal transfer and spread throughout hospital wards and rooms. Data indicate that patients admitted to a room previously occupied by someone with CDI have a 27% higher chance of being diagnosed with CDI if they were exposed within the last 90 days (odds ratio (OR) = 1.269). The risk increases to 40% (OR = 1.401) for those exposed in the last 365 days, even after accounting for previous admissions and length of stay. Additionally, the cumulative patient-day exposure to rooms that were once CDI-positive is also significant; each day of exposure within the last 90 days increases the odds of CDI by 4.5%, while exposure within the previous 365 days increases the odds by 4.2% [11].

Moreover, antimicrobial resistance is one of the key variables contributing to the present CDI epidemic and the spread of emerging strains, which involves several mechanisms and alterations in various components [12].

Over the past 30 years, the burden of CDI has steadily increased worldwide, particularly in areas with a high to moderate Simpson’s Diversity Index (SDI). Across many risk categories, there is a significant positive association between CDI and global antimicrobial consumption.

According to the CDC’s *Antibiotic Resistance Threats in the USA* report, CDI was responsible for about 223,900 hospitalized patient cases, 12,800 projected deaths, and $1 billion in medical expenses in 2017. According to a prospective point-prevalence multicenter study conducted in Europe, there were 7.0 CDI infections for every 10,000 hospital patient bed days. Additionally, other research revealed significantly different CDI death rates: attributable mortality at 30 days may range from 5.7 to 6.9%, while all-cause mortality at 30 days ranges from 9 to 38%, highlighting the significance of comprehending CDI’s epidemiological features [13,14].

*C. difficile* infections have a substantial impact on the EU/EEA population. The ECDC estimates that in 2016 and 2017, there were roughly 189,526 (approximate range: 105,000–341,000) cases of healthcare-associated *C. difficile* infection (HA CDI) in acute care hospitals annually, including an estimated 7419 (approximate range: 4100–13,300) fatal HA CDI cases per year with CDI as a possible contributing factor. From 2009 to 2013, HA CDI had the fourth highest burden of any single infectious disease under surveillance at the European level in terms of disability-adjusted life years (DALYs).

To better understand the epidemiology of *C. difficile* at the local, national, and European levels, the European Centre for Disease Prevention and Control (ECDC) began coordinating CDI surveillance in Europe, where some countries reported PCR ribotype and toxin production data. Croatia participated with 24 hospitals using a light surveillance protocol that excluded the genetic information of isolates. The mean HA CDI incidence density for Croatia in the ECDC survey was 2.37 cases per 10,000 patient days (PD) in 2016 and 3.01 in 2017 (the EU/EEA HA CDI mean was 2.87 and 1.91, respectively), established by the same methodology. In the other surveillance period of 2018 to 2020, the reported mean hospital incidence density for HA CDI was 2.58 cases per 10,000 PD in 2020, compared to 2.02 in 2019 and 2.79 in 2018. The median testing rate for the EU/EEA was 31.5 in 2016 and 49.2 in 2017 per 10,000 PD; for Croatia, it was 19.8 in 2016 and 27.8 in 2017 per 10,000 PD [15,16,17].

Determining phylogenetic relationships across geographic regions is crucial in reducing transmission rates and potential healthcare burden [10]. Standardized methods for estimation of the genetic relatedness and diversity of *C. difficile* strains include PCR ribotyping, multilocus sequence typing (MLST), pulsed-field gel electrophoresis (PFGE), and whole-genome sequencing (WGS), with varying representation worldwide [18,19].

Isolates collected from seven laboratories in Croatia during the 6-month period in 2016 were sent for PCR ribotyping that was performed in MUMC, Leiden, Netherlands. The most common ribotype among *C. difficile* isolates was type 176 (36%), followed by 027 (16%), 022, 077, etc. This was the first national study on the incidence of CDI and the molecular characteristics of *C. difficile* isolates in Croatia [20].

Although some data on the molecular epidemiology of *C. difficile* strains in Croatia are available, limited genetic research has been conducted, and no comprehensive studies using WGS for *C. difficile* characterization in Croatia have been conducted as of now [21,22,23].

In this study, we aimed to use WGS to characterize *C. difficile* strains isolated from patients hospitalized at University Hospital Centre (UHC) Zagreb. Our objective was to investigate strain distribution among our hospital population, research possible transmission pathways, and compare it to previously conducted studies on *C. difficile* in Croatia and other European countries to identify dominant strains.

Examining and applying WGS data may help map the evolution of significant strains, resolve relapses/reinfections in recurrent CDI, and evaluate hospital infection prevention and control (IPC) performance.

## 2. Materials and Methods

A total of 107 positive non-duplicate *C. difficile* stool samples were collected between 1 January 2022 and 31 December 2022 at the UHC Zagreb, the central and largest hospital in Croatia. After removing highly contaminated samples and samples from which assembly was impossible, 103 independent *C. difficile* samples were included in the study.

Testing for *C. difficile* was indicated in patients with symptoms consistent with CDI, based on the hospital algorithm and microbiology laboratory protocol. NAAT tests were used to detect GDH and toxins. Stools that tested positive were cultured, and *C. difficile* was isolated and preserved in the freezer at −80 °C until further testing.

The patients in the study had no prior CDIs in their medical history, and all the cases included in our study occurred during their recent hospital stays. Based on the ECDC definition, all the CDI cases were considered healthcare-acquired infections.

WGS was performed on all 103 independent *C. difficile* isolates by an ECDC laboratory contractor (Eurofins Genomics, Ebersberg, Germany) as a part of continuous surveillance by the ECDC. DNA extraction was carried out by Eurofins Genomics (Eurofins Genomics, Ebersberg, Germany) using silica membrane kits (Macherey & Nagel Food Kit, Düren, Germany) and RNase treatment after mechanical disruption. Next-generation sequencing libraries were prepared with the NEBNext Ultra II FS DNA Library Prep Kit (New England Biolabs, Frankfurt am Main, Germany) and sequenced on Illumina NovaSeq 6000 systems (Illumina Inc., San Diego, CA, USA) in the paired-end 150 bp mode.

Genomes were assembled using SKESA, which is included in the Ridom™ SeqSphere+ v.10.0.4 (Ridom GmbH, Münster, Germany) software. The sequences were analyzed by MLST and cgMLST using the SeqSphere+ (version 10.0.4) cgMLST scheme version 2.0 based on 2147 core genes and 1357 accessory genes. A limit of 95% good cgMLST target genes was set for inclusion in the cgMLST analysis. Genetic relationships among strains were visualized using the minimal spanning tree (MST) algorithm for 103 samples based on 3504 columns, pairwise and ignoring missing values, enabling the identification of genetically similar and distinct strains. Clusters were defined using a threshold of 3 allelic differences, which is proposed as the discriminatory threshold for outbreak detection [24].

The presence of toxin genes (*tcdA*, *tcdB*, *cdtA*, and *cdtB*) was analyzed using ABRicate (v.1.0.1) based on the Virulence Factor Database (VFDB) [25,26]. The presence of antimicrobial resistance genes was determined using the ResFinder function in ABRicate (v.1.0.1) based on the Resistance Gene Identifier and CARD databases [27].

The collected data were organized by patient information, testing results (toxins, genetic mutations, and antimicrobial resistance), and epidemiological information (cluster and sequence type). The patient information collected was basic demographic information (sex and age), current admission and stay information (patient’s ward), previous hospitalizations (at least 2 days in the last 90 days before recent hospitalization), recent antimicrobial usage (2 weeks before isolation of *C. difficile*), and presence of comorbidities linked to severe CDI and recurrent CDI (psychiatric disorders, gastroesophageal reflux disease, chronic obstructive pulmonary disease, cardiovascular disease, cerebrovascular disease, diabetes, malignancies, renal function impairment or failure, ulcerative colitis or other IBD, or immunocompromised status). The Charlson comorbidity index (CCI) was calculated for all patients included in the study based on the data collected. Isolates in the same cluster (under 3 allelic differences) were considered genetically linked. Patients who shared time (two positive samples collected within 28 days) on the same ward were considered to have ward contact [28].

## 3. Results

### 3.1. Patient Characteristics

**Figure 1:**
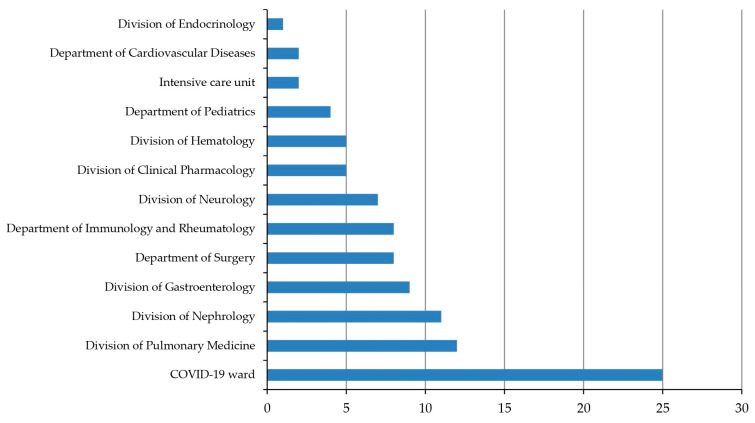
Ward distribution of *C. difficile* samples. Number of isolates.

Of the 103 independent *C. difficile* samples collected between 1 January 2022 and 31 December 2022, 100 (97.1%) isolates were identified as toxigenic *C. difficile* (TCD). Patient age varied from 0 to 93, with a median age of 73. The proportions of male and female patients were almost equal, at 49.5% and 50.5%, respectively (Table 1). Samples were collected from patients hospitalized in 13 wards (Figure 1). Most collected *C. difficile* samples come from the COVID-19 ward (25, 24.3%), followed by the Division of Pulmonary Medicine (12, 11.7%) and the Division of Nephrology (11, 10.7%).

### 3.2. Characterization of C. difficile Isolates by WGS

**Figure 2:**
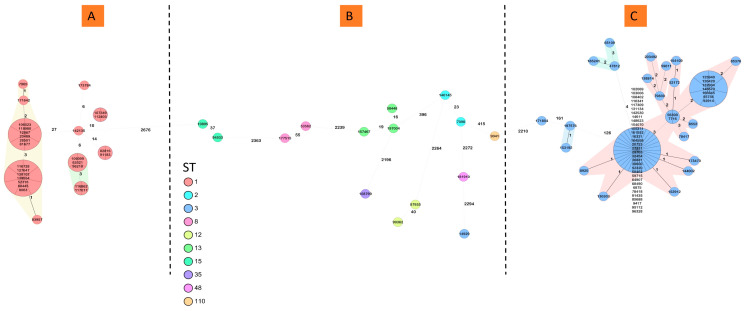
Genomic structure of the *C. difficile* samples. A minimum spanning tree of 103 *C. difficile* isolates was generated using Ridom SeqSphere+ based on 3504 columns (core and accessory genome), pairwise and ignoring missing values. Distance is based on columns from *C. difficile* cgMLST v2 (2147) and Accessory v2 (1357). The cluster distance threshold is 3 allelic differences. Color shading in the background is different in identified clusters. (**A**) ST1 isolates, comprising 4 distinct clusters, ST1-C1, ST1-C2, ST1-C3, ST1-C4, and two ST1 singletons. (**B**) ST2, ST8, ST12, ST13, ST15, ST35, ST48, and ST110 singletons. (**C**) ST3 isolates, comprising 3 distinct clusters, ST3-C5, ST3-C6, ST3-C7, and an ST3 singleton.

Most of the 103 *C. difficile* isolates were assigned to two predominant sequence types (ST), of which ST3 (62, 60.2%) comprised the most isolates, followed by ST1 (27, 26.2%). Other isolates were assigned to ST2, ST8, ST12, ST13, ST15, ST35, ST48, and ST110 (Table S1, Figure 2).

Virulence gene analysis showed that 97.1% (100/103) of isolates were TCD, including genotypes *tcdA + B + cdtA-B-* (70/100, 70%), *tcdA-B + cdtA + B +* (27/100, 27%), and *tcdA-B + cdtA-B-* (3/100, 3%) (Figure S1). Among TCD isolates, ST3 (61/100, 61%) was the most prevalent type, followed by ST1 (27/100, 27%), ST13 (3/100, 3%), ST12 (2/100, 2%), ST2 (2/100, 2%), ST8 (2/100, 2%), ST110 (1/100, 1%), ST48 (1/100, 1%), and ST35 (1/100, 1%). ST15 (2/3, 66.7%) was the most prevalent ST in nontoxigenic *C. difficile* (NTCD), with ST3 being the only ST containing both TCD (61/100, 61%) and NTCD (1/3, 33.3%) (Figure S2).

**Figure 3:**
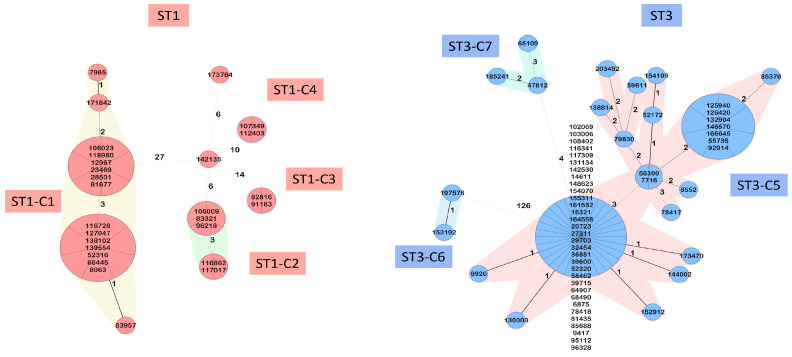
Genomic structure of the ST1 and ST3 clusters. A minimum spanning tree was generated using Ridom SeqSphere+. The distance based on columns from *C. difficile* cgMLST v2 (2147) and Accessory v2 (1357). The cluster distance threshold is 3 allelic differences. ST1 (ST1-C1, ST1-C2, ST1-C3, and ST1-C4) and ST3 (ST3-C5, ST3-C6, and ST3-C7) clusters were identified.

The MST algorithm with a cutoff of three allelic differences revealed seven distinct clusters. The clusters ranged in size from 2 to 55 isolates and corresponded to the branches of the phylogenetic tree (Table S1, Figure 2). ST1 comprised four distinct clusters, named ST1-C1 (sixteen isolates), ST1-C2 (five isolates), ST1-C3 (two isolates), and ST1-C4 (two isolates). The other identified clusters were all ST3: ST3-C5 (fifty-five isolates), ST3-C6 (two isolates), and ST3-C7 (three isolates) (Figure 3). The remaining 18 isolates were singletons.

**Figure 4:**
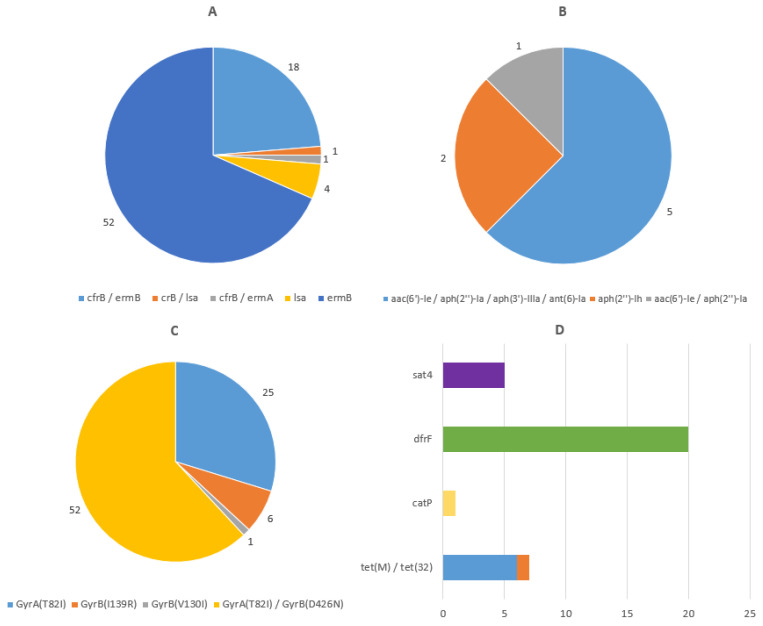
Distribution of antimicrobial resistance determinants (ARDs). Identified by the ResFinder and CARD databases, presented as the number of isolates. (**A**) Macrolide, lincosamide, and streptogramin B ARDs. (**B**) Aminoglycoside ARDs. (**C**) Fluoroquinolone ARDs. (**D**) Streptothricin, trimethoprim, chloramphenicol, and tetracycline ARDs.

The ResFinder and CARD databases have singled out a total of 18 antimicrobial resistance determinants (ARDs), which confer antimicrobial resistance to macrolides, lincosamide, streptogramin B, aminoglycosides, fluoroquinolones, chloramphenicol, trimethoprim, streptothricin, and tetracycline. Erythromycin ribosomal methylase genes of class B (*ermB*), the most common ARDs conferring resistance to the macrolide–lincosamide–streptogramin B (MLSB) family in our study, were detected in 68% (70/103) of isolates. Other MLSB ARDs found were *cfrB* in 19.4% (20/103), *lsa* in 4.9% (5/103) of isolates, and *ermA* in one isolate. Aminoglycoside resistance genes were found in 7.8% (8/103) of isolates, notably *aac(6′)-Ie*, *aph(2″)-Ia*, *aph(3′)-IIIa*, *ant(6)-Ia,* and *aph(2″)-Ih.* Amino acid substitutions in the DNA gyrases GyrA and GyrB are responsible for fluoroquinolone resistance in *C. difficile.* The detected substitutions in GyrA/GyrB were GyrA (T82I) in 74.8% (77/103), GyrB (D426N) in 50.5% (52/103), GyrB (I139R) in 5.8% (6/103) of isolates, and GyrB (V130I) in one of the isolates. The chloramphenicol resistance gene *catP* was detected in one of the isolates, the trimethoprim resistance gene *dfrF* in 19.4% (20/103), the streptothricin resistance gene *sat4* in 4.9% (5/103), and the tetracycline resistance gene *tet*(M) in 5.8% (6/103) of the isolates, with the tetracycline resistance gene *tet*(32) being found in one isolate (Figure 4).

### 3.3. Nosocomial Transmission

**Figure 5:**
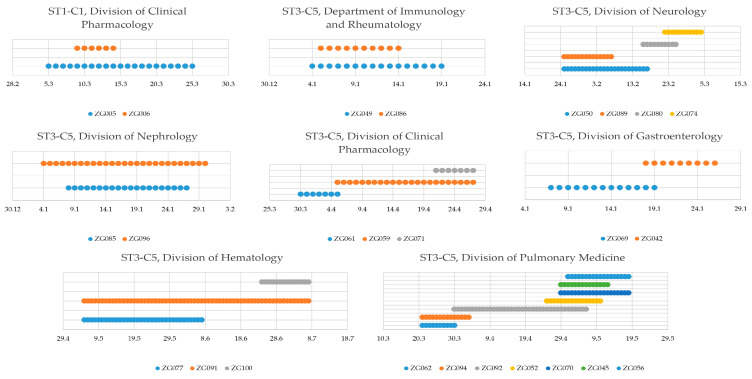
Nosocomial transmission information. Timeline of patients with genetically linked isolates. Cluster, patient ward, and time on ward indicated.

**Figure 6:**
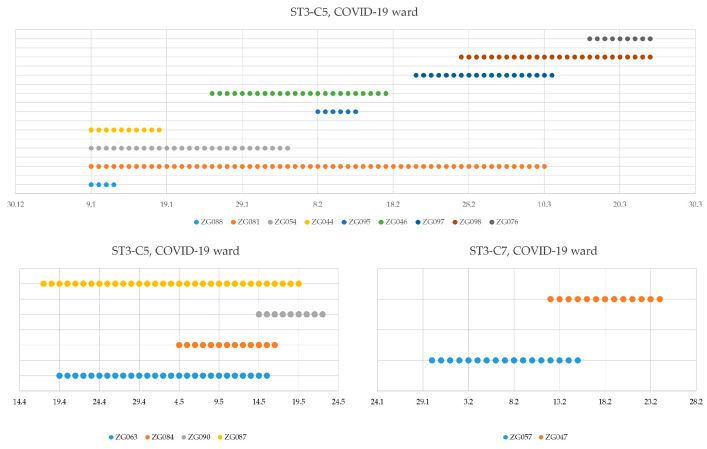
Nosocomial transmission in the COVID-19 ward. Timeline of patients with genetically linked isolates.

One of the more important routes of pathogen transmission is via nosocomial transmission. Of 103 *C. difficile* isolates collected between 1 January 2022 and 31 December 2022, 30 had a spatiotemporal concordance with a previously isolated *C. difficile* sample. We identified five genetically linked case pairs that had ward contact, namely, ST1-C1 (Division of Clinical Pharmacology), ST3-C5 (Department of Immunology and Rheumatology), ST3-C5 (Division of Nephrology), ST3-C7 (COVID-19 ward), and ST3-C5 (Division of Gastroenterology). Genetically linked triplets with ward contact were identified in the Division of Clinical Pharmacology and Division of Hematology, both assigned to ST3-C5. The Division of Neurology and Division of Pulmonary Medicine had four and seven isolates implicated in nosocomial transmission, respectively (Figure 5). The biggest occurrence of possible nosocomial transmission happened in the COVID-19 ward, with nine isolates sharing a genetic link and ward contact from 9 January 2022 to 24 March 2022, followed by another four isolates from 17 April 2022 to 22 May 2022, without patient-to-patient contact between the two groups. The aforementioned case was the only possible instance of ward contamination leading to nosocomial transmission that we identified (Figure 6).

## 4. Discussion

Over the last decade, there has been an increased interest in *C. difficile*. Its high mortality in certain populations and high relapse rates impose an increasing economic and health burden upon Europe [4,29]. The identification of hypervirulent strains in some areas of the world, with increased transmissibility and virulence, has resulted in outbreaks with relatively high fatality rates within facilities. In Croatia, most previous studies focusing on molecular characterization and possible nosocomial transmission have used ribotyping as a method [21,22,23]. In this study, we performed a comprehensive genomic analysis of *C. difficile* isolates from patient samples taken between 1 January 2022 and 21 December 2022 in the largest healthcare center in Croatia.

Most of the 103 *C. difficile* isolates were assigned to two predominant sequence types (ST), of which ST3 (62, 60.2%) comprised the most isolates, followed by ST1 (27, 26.2%).

Other isolates were assigned to ST2, ST8, ST12, ST13, ST15, ST35, ST48, and ST110.

If we compare our findings with the study of Rupnik et al., we can see that ST3 is predominant in isolates of human, hospital origin and in European countries with similarities to Croatia, namely, Italy, Romania, and Spain, and ST1 has also been present for quite some time in Romania, Austria, Poland, Italy, etc. The aforementioned study analyzed isolates collected in 2018. They identified various genetic backgrounds across Europe from different sources, including human sources, environment, food, and animals [30].

In the study of the genomic diversity of *C. difficile*, conclusions have been drawn from whole-genome comparisons based on single nucleotide polymorphism (SNP) analysis of the core genome, MLST, and comparative phylogenomics. They show that *C. difficile* evolved through various lineages, and six different phylogenetic clades, designated 1 through 5 and C-I, are described. In terms of PCR ribotypes and STs (>100 STs), Clade 1 has the greatest heterogeneity. Clade 1 contains the well-known PCR ribotypes 001 (ST-3), 012 (ST-54), and 014 (ST2, ST13, ST14, ST49, ST50, and ST132). A total of 18 distinct STs were discovered in clade 2, along with ST-1 (whose representative is the infamous PCR ribotype 027) [30].

To our knowledge, this is the first comprehensive genomic study utilizing WGS to investigate potential nosocomial transmission of *C. difficile* in a Croatian hospital.

Although the use of genomics to investigate hospital *C. difficile* outbreaks is well documented, most investigations have been conducted retrospectively, either over a predetermined time period or after an outbreak had grown to a scale that clearly increased the prevalence of CDI. Since there are no WGS services in our facility, we used the opportunity offered and financed by the ECDC to perform WGS in Eurofins laboratories.

Within the timeframe, we collected detailed epidemiological and clinical data to investigate possible nosocomial transmission routes. Usually, as soon as a patient with *C. difficile* is identified, immediate infection control measures are undertaken. IPC personnel are sent to assess ward personnel’s compliance with contact measure precautions, usage of sporicidal disinfectants, and hand washing. During the COVID-19 pandemic, patient placement conditions were different, and patient transfer to single rooms was difficult, if not impossible, due to limited resources.

In the collection of analyzed isolates, we identified the largest occurrence of possible nosocomial transmission in the COVID-19 ward, with nine isolates sharing a genetic link and ward contact from 9 January 2022 to 24 March 2022, followed by another three isolates from the same cluster (ST3-C5) from 17 April 2022 to 22 May 2022, without patient-to-patient contact between the two groups. The aforementioned case was the only possible instance of ward contamination leading to nosocomial transmission we identified, possibly due to suboptimal ward conditions, as well as the impossibility of emptying and thoroughly cleaning the wards(s) after the identification of *C. difficile* isolates. There was a possible nosocomial transmission occurrence in the COVID-19 ward, with another two isolates from another cluster (ST3-C7) identified as well.

We also concluded that the Division of Neurology and the Division of Pulmonary Medicine had four and seven isolates implicated in nosocomial transmission, respectively.

Genetic analysis, together with epidemiological data, identified genetically linked triplets with ward contact in the Division of Clinical Pharmacology and the Division of Hematology, both of which were assigned to ST3-C5. In other wards, four genetically linked case pairs had been linked epidemiologically in the same ward with shared medical staff, namely, ST1-C1 (Division of Clinical Pharmacology), ST3-C5 (Department of Immunology and Rheumatology), ST3-C5 (Division of Nephrology), and ST3-C5 (Division of Gastroenterology).

COVID-19 wards were at times crowded, and double gloving was practiced. Inadequate use of PPE and empirical antimicrobial therapies for infections in hospitalized COVID-19 patients may raise the risk of CDI and antibiotic-associated diarrhea (AAD). Depletion of helpful commensals and enabling transmission of opportunistic pathogens such as *C. difficile* can also be linked to changes in the gut microbiota [31].

While young children have a high rate of *C. difficile* carriage, old age is considered a risk factor for CDI [32,33]. Our results confirm the aforementioned, as most of the CDI patients presented in this study were in the older age group. This suggests closer monitoring of such patients in hospital wards to ensure adequate and timely diagnosis of CDI.

*C. difficile* is frequently transmitted around the world, which has an impact on CDI epidemics. The dominant sequence type in Europe seems to be ST1, while other continents harbor ST11, ST3, and ST35 as prevalent types [34,35]. The most prevalent type in our study was ST3, followed by ST1, showing a shift in type prevalence. Most of the isolates, 97%, were toxigenic, with ST3 isolates making up the majority. Several studies have shown the impact of *tcdA* and *tcdB* variants on the diagnosis and disease severity [36,37]. Among our TCD isolates, 70% of isolates harbored *tcdA* and *tcdB*, while 97% of TCD isolates had at least one of the variants. Toxin variants and MLST had a good concordance rate in our study, indicating stability in our *C. difficile* isolates. Studies have shown frequent mutations in those genes; therefore, continuous monitoring is warranted in future studies [38].

Antimicrobial resistance genes found in isolates from our study showed a great propensity toward fluoroquinolone and macrolide–lincosamide–streptogramin B resistance, concordant with most studies covering *C. difficile* resistance [39,40]. No occurrence of vancomycin resistance genes was noted in our study. Potential implications of the presence of genes encoding antimicrobial resistance to macrolides, lincosamide, streptogramin B, aminoglycosides, fluoroquinolones, chloramphenicol, trimethoprim, streptothricin, and tetracycline should not have an impact on empirical treatment of CDIs, since current guidelines propose the use of vancomycin, fidaxomicin, and, in exceptional cases, metronidazole [41].

Possible nosocomial transmission cases were shown in eight of thirteen (61.5%) hospital wards. It shows a need for constant and vigilant monitoring of new and old CDI cases to prevent hospital-based horizontal transmission between patients. An especially strong case can be made for the COVID-19 ward, which not only had the highest rate of transmission cases out of all of the wards but was the only one that showed a possible ward-contamination-linked nosocomial transmission. Close monitoring of hospital wards that are vulnerable, whether due to patient characteristics or staffing issues, should be conducted to prevent nosocomial transmissions. Particular attention should be given to patients with diarrhea, ensuring that stool samples are sent for analysis and that the patients are accommodated in single-occupancy rooms with separate sanitary facilities. Cleaning protocols should be intensified, especially after the discharge of a *C. difficile* positive patient. It is necessary to verify the effectiveness of cleaning in the environment, on shared objects and instruments used on positive patients. Particular emphasis should be placed on hand hygiene of ward personnel.

This study had the following limitations: It was conducted over one year, painting a limited picture of a local epidemiological situation concerning the proportions of sequence types. Antimicrobial resistance could only be proposed from the presence of antimicrobial resistance genes because antimicrobial susceptibility testing was not conducted. Antimicrobial susceptibility testing was not performed according to the internal protocol of the microbiological laboratory, which utilizes NAAT and immunochromatographic methods for the detection of *C. difficile* to ensure rapid identification of toxigenic strains and provide information to the department for the implementation of appropriate antimicrobial therapy for patients and real-time infection control measures.

In this study, only the symptomatic patients were tested and included, meaning that the asymptomatic carriers, their role in transmission, and their impact on the genomic characteristics of collected isolates were missed.

Further research should include samples of close contacts, even if asymptomatic, improving the discovery of nosocomial transmission routes. Additionally, compliance with IPC measures, hand hygiene, and audits with scoring should be recorded, together with occasional environmental sampling, especially for high-touch surfaces.

## 5. Conclusions

Given the high antimicrobial resistance; high proportion of toxigenic *C. difficile* isolates; and diverse, unexpected genome characteristics among the *C. difficile* isolates at the UHC Zagreb, molecular epidemiological surveillance should be prioritized. With WGS becoming more readily available, a powerful tool such as this should be established as part of a local surveillance system to prevent nosocomial transmission and outbreaks of *C. difficile* infections.

By utilizing the powerful tool of real-time WGS, it would be possible to identify isolates with high epidemic potential, allowing infection control measures to be implemented earlier and more intensively, considering the potential risk of bacterial spore dissemination.

There is always room for improvement in hospital practices regarding testing, notifying the presence of *C. difficile*, and the timely use of appropriate antimicrobial therapy. One of the driving factors for CDI is the inappropriate and uncontrolled use of antimicrobials for the treatment of other infections and for prophylactic purposes. There should be a restrictive prescribing policy to prevent the emergence of antimicrobial resistance and occurrence of CDIs in patients.

## Figures and Tables

**Table 1:** Patient characteristics.

	Total CD Positive	TCD Positive
**Number of cases, N (%)**	103	100 (97.1%)
**Age, years** Mean ± SD Range Median [quartiles]	68.76 ± 21.03 0–93 73 [64.5, 83]	70.02 ± 18.99 0–93 73 [65.5, 83]
**Sex, N (%)** Female Male **Wards, N (%)**	52 51	50 (96.1%) 50 (98.0%)
COVID-19 ward	25	25 (100.0%)
Division of Pulmonary Medicine	12	12 (100.0%)
Division of Nephrology	11	11 (100.0%)
Division of Gastroenterology	9	9 (100.0%)
Department of Surgery	8	8 (100.0%)
Immunology and Rheumatology	8	7 (87.5%)
Division of Neurology	7	7 (100.0%)
Division of Clinical Pharmacology	5	5 (100.0%)
Division of Hematology	5	5 (100.0%)
Department of Pediatrics	4	2 (50.0%)
Other wards	9	9 (100.0%)
**Recent previous hospitalizations**	94	91 (96.8%)
**Recent antibiotic use**	81	80 (98.8%)
**Presence of comorbidities, N(%)**		
Psychiatric disorders	17	17 (100.0%)
Gastroesophageal reflux disease	5	5 (100.0%)
Chronic obstructive pulmonary disease	12	12 (100.0%)
Cardiovascular disease	31	30 (96.8%)
Cerebrovascular disease	12	12 (100.0%)
Diabetes	21	21 (100.0%)
Solid tumor	19	19 (100.0%)
Renal impairment or failure	20	20 (100.0%)
Ulcerative colitis	1	1 (100.0%)
Immunocompromised status	34	33 (97.1%)
**Charlson comorbidity index (≥5)**	58	57 (98.3%)

## Data Availability

The original contributions presented in this study are included in the article/Supplementary Material. Further inquiries can be directed to the corresponding author. All raw sequencing reads from the 103 *C. difficile* sequenced in this project were submitted to the European Nucleotide Archive (https://www.ebi.ac.uk/ena/; accessed on 13 November 2024) under Project Number PRJEB82445.

## Supplementary Materials

The following supporting information can be downloaded at: https://www.mdpi.com/article/10.3390/microorganisms12122434/s1. Figure S1. Toxin gene distribution among toxigenic *C. difficile* isolates. Figure S2. Toxigenic *C. difficile* (TCD) and non-toxigenic *C. difficile* (NTCD) distribution. Table S1. Genomic characteristics of isolates.

## References

[1] Lawson P.A., Citron D.M., Tyrrell K.L., Finegold S.M. (2016). Reclassification of Clostridium difficile as Clostridioides difficile (Hall and O’Toole 1935) Prévot 1938. Anaerobe.

[2] Finn E., Andersson F.L., Madin-Warburton M. (2021). Burden of Clostridioides difficile infection (CDI)—A systematic review of the epidemiology of primary and recurrent CDI. BMC Infect. Dis..

[3] Son K.J., Kim Y.A., Park Y.S. (2022). Economic burden attributable to Clostridioides difficile infections in South Korea: A nationwide propensity score-matched study. J. Hosp. Infect..

[4] Wingen-Heimann S.M., Davies K., Viprey V.F., Davis G., Wilcox M.H., Vehreschild M.J.G.T., Lurienne L., Bandinelli P.-A., Cornely O.A., Vilken T. (2023). COMBACTE-CDI consortium. Clostridioides difficile infection (CDI): A pan-European multicenter cost and resource utilization study, results from the Combatting Bacterial Resistance in Europe CDI (COMBACTE-CDI). Clin. Microbiol. Infect..

[5] Braae U.C., Møller F.T., Ibsen R., Ethelberg S., Kjellberg J., Mølbak K. (2020). The Economic Burden of *Clostridioides difficile* in Denmark: A Retrospective Cohort Study. Front. Public Health.

[6] Pereira J.A., McGeer A., Tomovici A., Selmani A., Chit A. (2020). Incidence and economic burden of *Clostridioides difficile* infection in Ontario: A retrospective population-based study. CMAJ Open.

[7] Fu Y., Luo Y., Grinspan A.M. (2021). Epidemiology of community-acquired and recurrent *Clostridioides difficile* infection. Ther. Adv. Gastroenterol..

[8] Viprey V.F., Davis G.L., Benson A.D., Ewin D., Spittal W., Vernon J.J., Rupnik M., Banz A., Allantaz F., Cleuziat P. (2022). COMBACTE-CDI National Coordinators; Wilcox MH, Davies KA.; COMBACTE-CDI consortium; Members of the COMBACTE-CDI National coordinators. A point-prevalence study on community and inpatient *Clostridioides difficile* infections (CDI): Results from Combatting Bacterial Resistance in Europe CDI (COMBACTE-CDI), July to November 2018. Euro Surveill..

[9] Buddle J.E., Fagan R.P. (2023). Pathogenicity and virulence of *Clostridioides difficile*. Virulence.

[10] Zhao H., Nickle D.C., Zeng Z., Law P.Y.T., Wilcox M.H., Chen L., Peng Y., Meng J., Deng Z., Albright A. (2021). Global Landscape of Clostridioides Difficile Phylogeography, Antibiotic Susceptibility, and Toxin Polymorphisms by Post-Hoc Whole-Genome Sequencing from the MODIFY I/II Studies. Infect. Dis. Ther..

[11] Sood G., Truelove S., Dougherty G., Landrum B.M., Qasba S., Patel M., Miller A., Wilson C., Martin J., Sears C. (2022). Clostridioides difficile infection (CDI) in a previous room occupant predicts CDI in subsequent room occupants across different hospital settings. Am. J. Infect. Control..

[12] Spigaglia P. (2016). Recent advances in the understanding of antibiotic resistance in *Clostridium difficile* infection. Ther. Adv. Infect. Dis..

[13] Chen Y., Xie X., Ge Q., He X., Sun Z., Li Y., Guo Y., Geng C., Li X., Wang C. (2024). The global burden and trend of Clostridioides difficile and its association with world antibiotic consumption, 1990–2019. J. Glob. Health.

[14] Balsells E., Shi T., Leese C., Lyell I., Burrows J., Wiuff C., Campbell H., Kyaw M.H., Nair H. (2019). Global burden of Clostridium difficile infections: A systematic review and meta-analysis. J. Glob. Health.

[15] European Centre for Disease Prevention and Control (2022). Clostridioides (Clostridium) Difficile Infections. Annual Epidemiological Report for 2016–2017.

[16] European Centre for Disease Prevention and Control (2024). Clostridioides Difficile Infections. Annual Epidemiological Report for 2018–2020.

[17] European Centre for Disease Prevention and Control (2024). Study Protocol for a Survey of Whole Genome Sequencing of Clostridioides Difficile Isolates from Tertiary Acute Care Hospitals, EU/EEA, 2022–2023.

[18] Martínez-Meléndez A., Morfin-Otero R., Villarreal-Treviño L., Baines S.D., Camacho-Ortíz A., Garza-González E. (2020). Molecular epidemiology of predominant and emerging Clostridioides difficile ribotypes. J. Microbiol. Methods.

[19] Abad-Fau A., Sevilla E., Martín-Burriel I., Moreno B., Bolea R. (2023). Update on Commonly Used Molecular Typing Methods for Clostridioides difficile. Microorganisms.

[20] Budimir A., Mareković I., Mijač M., Bosnjak Z., Payerl-Pal M., Susic E., Matas I., Novak A., Harmanus C., Kuijper E. (2018). Epidemiology of *Clostridium difficile* Infections in Croatia-National Study. CROSBI. https://www.bib.irb.hr:8443/1238985.

[21] Paradžik M. (2017). The First Evidence of Epidemic Strain Clostridium Difficile (027/NAP1/BI) in Eastern Croatia. J. Clin. Microbiol. Biochem. Technol..

[22] Novak A., Spigaglia P., Barbanti F., Goic-Barisic I., Tonkic M. (2014). First clinical and microbiological characterization of Clostridium difficile infection in a Croatian University Hospital. Anaerobe.

[23] Rupnik M., Andrasevic A.T., Dokic E.T., Matas I., Jovanovic M., Pasic S., Kocuvan A., Janezic S. (2016). Distribution of Clostridium difficile PCR ribotypes and high proportion of 027 and 176 in some hospitals in four South Eastern European countries. Anaerobe.

[24] Baktash A., Corver J., Harmanus C., Smits W.K., Fawley W., Wilcox M.H., Kumar N., Eyre D.W., Indra A., Mellmann A. (2022). Comparison of Whole-Genome Sequence-Based Methods and PCR Ribotyping for Subtyping of *Clostridioides difficile*. J. Clin. Microbiol..

[25] Zankari E., Allesøe R., Joensen K.G., Cavaco L.M., Lund O., Aarestrup F.M. (2020). PointFinder: A novel web tool for WGS-based detection of antimicrobial resistance associated with chromosomal point mutations in bacterial pathogens. J. Antimicrob. Chemother..

[26] Camacho C., Coulouris G., Avagyan V., Ma N., Papadopoulos J., Bealer K., Madden T.L. (2009). BLAST+: Architecture and applications. BMC Bioinform..

[27] Alcock B.P., Raphenya A.R., Lau T.T.Y., Tsang K.K., Bouchard M., Edalatmand A., Huynh W., Nguyen A.-L.V., Cheng A.A., Liu S. (2020). CARD 2020: Antibiotic resistome surveillance with the comprehensive antibiotic resistance database. Nucleic Acids Res..

[28] Wen X., Shen C., Xia J., Zhong L.-L., Wu Z., Ahmed M.A.E.-G.E.-S., Long N., Ma F., Zhang G., Wu W. (2022). Whole-Genome Sequencing Reveals the High Nosocomial Transmission and Antimicrobial Resistance of Clostridioides difficile in a Single Center in China, a Four-Year Retrospective Study. Microbiol. Spectr..

[29] Reigadas E., Vázquez-Cuesta S., Bouza E. (2024). Economic Burden of Clostridioides difficile Infection in European Countries. Adv. Exp. Med. Biol..

[30] Rupnik M., Viprey V., Janezic S., Tkalec V., Davis G., Sente B., Devos N., Muller B.H., Santiago-Allexant E., Cleuziat P. (2024). Distribution of *Clostridioides difficile* ribotypes and sequence types across humans, animals and food in thirteen European countries. Emerg. Microbes Infect..

[31] Azimirad M., Noori M., Raeisi H., Yadegar A., Shahrokh S., Asadzadeh Aghdaei H., Bentivegna E., Martelletti P., Petrosillo N., Zali M.R. (2021). How Does COVID-19 Pandemic Impact on Incidence of Clostridioides difficile Infection and Exacerbation of Its Gastrointestinal Symptoms?. Front. Med..

[32] Leffler D.A., Lamont J.T. (2015). Clostridium difficile infection. N. Engl. J. Med..

[33] Enoch D.A., Butler M.J., Pai S., Aliyu S.H., Karas J.A. (2011). Clostridium difficile in children: Colonisation and disease. J. Infect..

[34] Freeman J., Bauer M.P., Baines S.D., Corver J., Fawley W.N., Goorhuis B., Kuijper E.J., Wilcox M.H. (2010). The changing epidemiology of Clostridium difficile infections. Clin. Microbiol. Rev..

[35] Liu X.S., Li W.G., Zhang W.Z., Wu Y., Lu J.X. (2018). Molecular Characterization of Clostridium difficile Isolates in China From 2010 to 2015. Front. Microbiol..

[36] Dingle K.E., Griffiths D., Didelot X., Evans J., Vaughan A., Kachrimanidou M., Stoesser N., Jolley K.A., Golubchik T., Harding R.M. (2011). Clinical Clostridium difficile: Clonality and pathogenicity locus diversity. PLoS ONE.

[37] Lanis J.M., Heinlen L.D., James J.A., Ballard J.D. (2013). Clostridium difficile 027/BI/NAP1 encodes a hypertoxic and antigenically variable form of TcdB. PLoS Pathog..

[38] Shen E., Zhu K., Li D., Pan Z., Luo Y., Bian Q., He L., Song X., Zhen Y., Jin D. (2020). Subtyping analysis reveals new variants accelerated evolution of Clostridioides difficile toxin, B. Commun. Biol..

[39] Zaiss N.H., Witte W., Nübel U. (2010). Fluoroquinolone resistance and Clostridium difficile, Germany. Emerg. Infect. Dis..

[40] Li H., Li W.-G., Zhang W.-Z., Yu S.-B., Liu Z.-J., Zhang X., Wu Y., Lu J.-X. (2019). Antibiotic resistance of clinical isolates of Clostridioides difficile in China and its association with geographical regions and patient age. Anaerobe.

[41] Bishop E.J., Tiruvoipati R. (2022). Management of *Clostridioides difficile* Infection in Adults and Challenges in Clinical Practice: Review and Comparison of Current IDSA/SHEA, ESCMID and ASID Guidelines. J. Antimicrob. Chemother..

