# Supplementary material for: Whole-Genome Sequencing-Based Characterization of Clostridioides difficile Infection Cases at the University Hospital Centre Zagreb
Source: Microorganisms. 2024 Nov 27;12(12):2434. doi: 10.3390/microorganisms12122434 (PMC11676685; doi:10.3390/microorganisms12122434)
Supplement: Supplementary file 1 [file microorganisms-12-02434-s001.zip › microorganisms-3321476-supplementary.pdf]

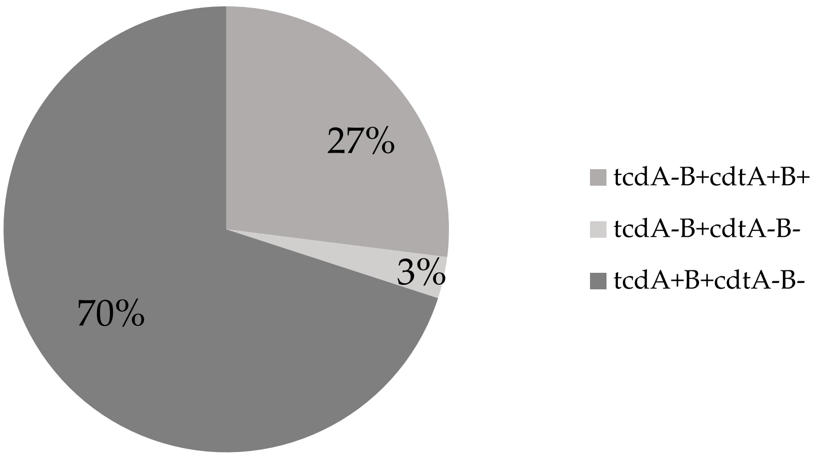

Figure S1. Toxin gene distribution among toxigenic *C. difficile* isolates.

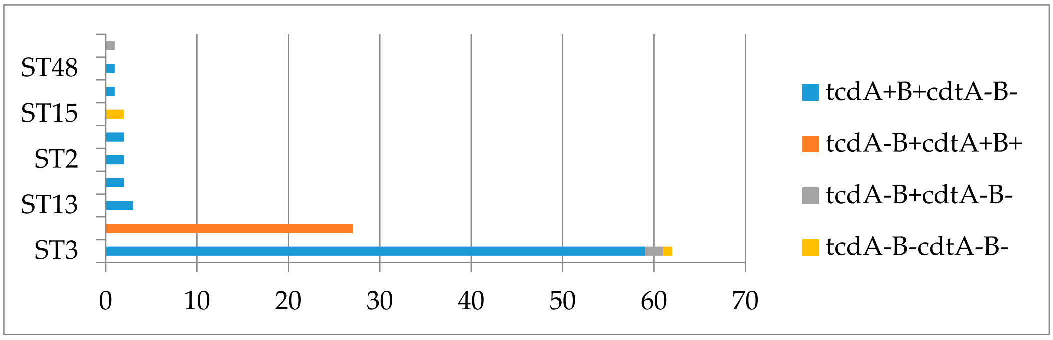

Figure S2. Toxigenic *C. difficile* (TCD) and non-toxigenic *C. difficile* (NTCD) distribution.

Table S1. Genomic characteristics of isolates.

Sample ID	Alias ID	ST	Cluster	Genome Size	cgMLST (%)		Sample ID	Alias ID	ST	Cluster	Genome Size	cgMLST (%)
52316	ZG001	1	ST1-C1	4.1	99.6		144002	ZG052	3	ST3-C5	4.1	99.8
28501	ZG002	1	ST1-C1	6.2	99.6		39600	ZG053	3	ST3-C5	4.1	99.9
138102	ZG003	1	ST1-C1	4.1	99.6		36881	ZG054	3	ST3-C5	4.1	99.9
171642	ZG004	1	ST1-C1	4.1	99.6		164558	ZG055	3	ST3-C5	4.2	99.9
116728	ZG005	1	ST1-C1	4.1	99.6		146570	ZG056	3	ST3-C5	4.1	99.8
83957	ZG006	1	ST1-C1	4.1	99.6		47812	ZG057	3	ST3-C7	4.1	99.8
106023	ZG007	1	ST1-C1	4.1	99.6		126420	ZG058	3	ST3-C5	4.1	99.9
7965	ZG008	1	ST1-C1	5.8	99.6		85376	ZG059	3	ST3-C5	7.3	99.9
23469	ZG009	1	ST1-C1	7.1	99.6		6875	ZG060	3	ST3-C5	4.1	99.9
68445	ZG010	1	ST1-C1	4.1	99.6		92914	ZG061	3	ST3-C5	4.1	99.9
12967	ZG011	1	ST1-C1	4.1	99.5		96328	ZG062	3	ST3-C5	4.1	99.9
139554	ZG012	1	ST1-C1	4.1	99.6		154070	ZG063	3	ST3-C5	4.1	99.9
118980	ZG013	1	ST1-C1	4.1	99.6		58462	ZG064	3	ST3-C5	4.1	99.9
8063	ZG014	1	ST1-C1	4.1	99.6		125940	ZG065	3	ST3-C5	4.1	99.9
127047	ZG015	1	ST1-C1	4.1	99.6		79830	ZG066	3	ST3-C5	4.1	99.9
81677	ZG016	1	ST1-C1	4.1	99.6		152912	ZG067	3	ST3-C5	4.1	99.9
116862	ZG017	1	ST1-C2	4.1	99.8		131134	ZG068	3	ST3-C5	4.1	99.9
83321	ZG018	1	ST1-C2	4.1	99.8		14611	ZG069	3	ST3-C5	4.1	99.9

96218	ZG019	1	ST1-C2	4.1	99.8		161552	ZG070	3	ST3-C5	4.1	99.9
106009	ZG020	1	ST1-C2	4.1	99.8		132904	ZG071	3	ST3-C5	4.1	99.8
117017	ZG021	1	ST1-C2	4.1	99.8		166645	ZG072	3	ST3-C5	4.1	99.9
142135	ZG022	1	singleton	4.1	99.8		27311	ZG073	3	ST3-C5	4.1	99.9
173784	ZG023	1	singleton sisingleton	4.1	99.8		68490	ZG074	3	ST3-C5	4.1	99.9
82816	ZG024	1	ST1-C3	4.1	99.8		95112	ZG075	3	ST3-C5	4.1	99.9
91183	ZG025	1	ST1-C3	4.1	99.8		103006	ZG076	3	ST3-C5	4.1	99.9
107349	ZG026	1	ST1-C4	4.1	99.8		154109	ZG077	3	ST3-C5	4.1	99.9
112403	ZG027	1	ST1-C4	4.2	99.8		117309	ZG078	3	ST3-C5	7.3	99.9
10895	ZG028	15	singleton	4.2	99.8		64907	ZG079	3	ST3-C5	4.1	99.9
94533	ZG029	15	singleton	4.1	99.8		59611	ZG080	3	ST3-C5	4.1	99.9
177515	ZG030	8	singleton	5.4	99.9		8920	ZG081	3	ST3-C5	4.1	99.9
53562	ZG031	8	singleton	4.2	99.9		78418	ZG082	3	ST3-C5	4.1	99.9
146145	ZG032	2	singleton	4.2	100.0		85688	ZG083	3	ST3-C5	4.1	99.9
7098	ZG033	2	singleton	4.0	100.0		148623	ZG084	3	ST3-C5	4.1	99.9
87935	ZG034	12	singleton	4.1	99.9		9417	ZG085	3	ST3-C5	4.1	99.9
99362	ZG035	12	singleton	4.1	99.8		7716	ZG086	3	ST3-C5	4.1	99.9
58448	ZG036	13	singleton	4.2	100.0		155311	ZG087	3	ST3-C5	7.0	99.9
157467	ZG037	13	singleton	4.2	100.0		8552	ZG088	3	ST3-C5	4.1	99.9
197004	ZG038	13	singleton	4.2	100.0		32454	ZG089	3	ST3-C5	7.4	99.7
108799	ZG039	35	singleton	4.2	99.5		173470	ZG090	3	ST3-C5	4.1	99.9
181910	ZG040	48	singleton	4.0	99.9		138814	ZG091	3	ST3-C5	4.1	99.9
9041	ZG041	110	singleton	4.1	99.7		116341	ZG092	3	ST3-C5	4.1	99.9
16321	ZG042	3	ST3-C5	7.3	99.9		55735	ZG093	3	ST3-C5	4.1	99.8
59715	ZG043	3	ST3-C5	4.1	99.9		102069	ZG094	3	ST3-C5	4.1	99.9
20723	ZG044	3	ST3-C5	4.1	99.9		52320	ZG095	3	ST3-C5	4.1	99.9
142530	ZG045	3	ST3-C5	4.1	99.9		29703	ZG096	3	ST3-C5	7.3	99.9
52172	ZG046	3	ST3-C5	4.1	99.9		78417	ZG097	3	ST3-C5	4.1	99.8
65109	ZG047	3	ST3-C7	4.1	99.9		81435	ZG098	3	ST3-C5	4.1	99.9
130303	ZG048	3	ST3-C5	4.1	99.9		185241	ZG099	3	ST3-C7	4.1	99.8
14929	ZG049	3	ST3-C5	4.1	99.9		203492	ZG100	3	ST3-C5	4.1	99.7
56300	ZG050	3	ST3-C5	4.1	99.9		153192	ZG101	3	ST3-C6	4.1	99.9
108402	ZG051	3	ST3-C5	4.1	99.9		197576	ZG102	3	ST3-C6	4.1	99.9
							171804	ZG103	3	singleton	4.3	99.8
